# Study of Laser Light Scattering Methods in Rapid Viability Assessment of Microorganisms under Antibiotics Exposure for Adaptation in Lab-on-A-Chip Format

**DOI:** 10.3390/diagnostics13061130

**Published:** 2023-03-16

**Authors:** Tatiana M. Zimina, Olga A. Pinchuk, Dmitry I. Kaplun, Lyudmila A. Kraeva, Nikita O. Sitkov

**Affiliations:** 1 Department of Micro and Nanoelectronics, Saint Petersburg Electrotechnical University “LETI”, 197022 Saint Petersburg, Russia; 2 The D.I. Mendeleev All-Russian Institute for Metrology (VNIIM), 190005 Saint Petersburg, Russia; 3 Department of Automation and Control Processes, Saint Petersburg Electrotechnical University “LETI”, 197022 Saint Petersburg, Russia; 4 Saint Petersburg Pasteur Institute, 197101 Saint Petersburg, Russia

**Keywords:** antibiotic resistance, antimicrobial susceptibility testing, viability analysis, microorganisms, laser light scattering, *Escherichia coli*, ceftriaxone

## Abstract

The antibiotic resistance (ABR) problem is becoming increasingly disturbing and it is important to implement express methods of ABR testing to allow operative antibiotic therapy decisions. The application of laser light scattering (LLS) in microbiological analysis for express ABR testing of microorganisms has been considered. The ways of miniaturization of laser light scattering for creating the bases of their integration into microbiological laboratory-on-a-chip (MLOC) for clinical express diagnostics have been analysed. The advantage of miniaturization in the context of clinical express analysis realization problems are investigated. A system of parallel measuring cells and illumination, enabling simultaneous testing of a group of antibiotics, was tested by splitting a laser beam with a two-dimensional collimator prepared of nanoporous anodic aluminum oxide. It has been demonstrated that the application of LLS methods, providing high concentration and mass sensitivity as well as a miniaturization potential, is an effective approach in the development of new generation diagnostic instruments. The studies have demonstrated the ability of methods to register effects of antibiotics on microbiological samples within 10 min. The following microorganisms were used in the study: *Escherichia coli* M-17, *Lactobacillus plantarum*, *Bifidobacterium bifidum*, *Stenotrophomonas maltophilia*.

## 1. Introduction

In recent years, the antibiotic resistance (ABR) of microorganisms has become an increasingly disturbing problem [1]. As a result, the availability of ABR rapid diagnostic tools for making the right decisions on the treatment of infectious diseases has become extremely important. The modern state-of-the-art methods in ABR testing are analysed in contemporary reviews [2,3], where a number of main methods used in this field are described. However, all of the methods were referred to as time-consuming. The reviews show that both the culture-based (phenotypic) and the genetic methods need 1–3 days, which does not meet modern requirements for prompt decision-making on treatment tactics. Thus, to date, there are no express and reliable methods for ABR testing, as demonstrated in recent reviews [2,3].

The European Committee on Antimicrobial Susceptibility Testing—AST (EUCAST) recommends, within the scope of various ABR testing methods, using phenotypic evaluation, i.e., experimental procedures to monitor the microbial growth in the presence of antibiotics, to achieve reliable ABR results [4]. EUCAST notes that culture-based ASTs require from 24 to 72 h and that this time lapse can be crucial for the patient [5,6,7].

Alternative approaches that have elevated AST speed over the past 25 years, such as nucleic acid-mediated amplification technologies, proteomics, next-generation sequencing, etc., have not yet lead to major changes in the routine high-throughput clinical microbiological detection of ABR [8].

**Figure 1:**
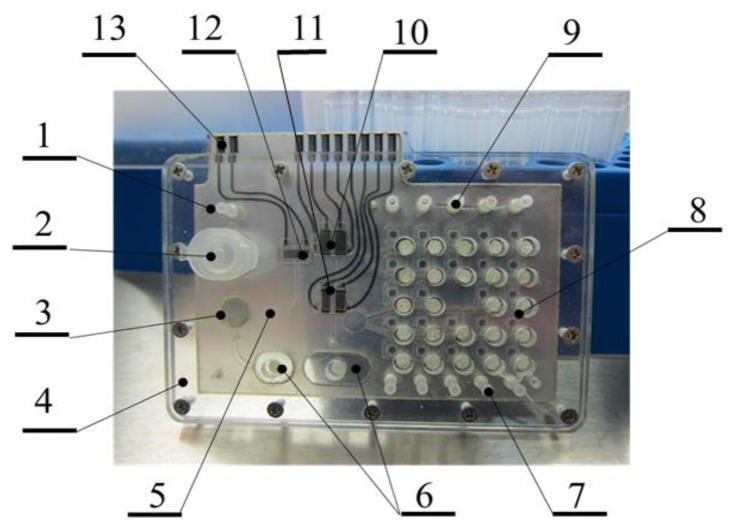
A sample model of lab-on-a-chip for total microbiological analysis. 1—pump inlet; 2—schwab inlet well; 3—growth chamber; 4—case; 5—CCD image capture area; 6—waste basins; 7, 9—antibiotic injection sleeves; 8—bacteria viability testing cells (two series of 10 cells); 10—impedimetric sensor; 11—SAW keys; 12—acoustic sensor; and 13—contact pads [9].

A prospective approach, which could be realized due to achievements of modern flexible electronic technologies, is the development towards hybrid-integrated laboratories-on-a-chip for total microbiological analysis—MLOC [9,10]. This approach enables realization on a single platform, an express growth of juvenile colonies of up to 1000 CFU in size during 4 h [9], and their identification and AST (Figure 1) [10].

The ABR stage on MLOC can be completed in a number of ways. Thus, the results can be expressed as the lowest concentration of the antibiotic that prevents visible growth, i.e., the minimal inhibitory concentration (MIC) [3]. Another way is the registration of microorganisms’ viability under antibiotic exposure [9,10]. Thus, it is possible to state that phenotypic methods realized on MLOC platforms, using various physical principles for microorganisms’ viability analysis, being direct and reliable, remain actual, provided that they are implemented in their express form and are accessible to people of all income levels [9,10]. Furthermore, the phenotypic approaches, recommended by experts [4], implemented in a lab-on-a-chip format can provide advantages of both the reliability of ABR determination and speed [9,10].

As mentioned previously, MLOC can be equipped with sensor elements based on various physical or chemical principles [10,11]. One of the prospective methods for bacteria mobility registration is laser light scattering (LLS), which is not invasive but is highly sensitive [12,13,14] and possesses miniaturization potential, particularly taking into account the new technologies and element base of solid-state electronics [15]. In regards to the sampling and culture growth, the use of hybrid devices is particularly promising since they can provide a sample of approximately 400–900 juvenile colonies [9,10], which may cover, for example, the complex anatomy of the human nose [16] or mouth [17] and might offer distinct microbial niches to be recognized and discriminated from the pathogens [13].

In this paper, we analysed the experimental data on the application of LLS in AST procedures, particularly in regards to the analysis time, sensitivity, and scaling potential for integration into hybrid MLOC.

Isolated attempts at LLS analysis of ABR were carried out back in the 1970s [18,19,20], which demonstrated the potential of the LLS method for express analysis of the lag period and the doubling time, along with the sensitivity of this method.

Later, laser light scattering was applied specifically in the field of AST, for example to study the effects of streptomycin on *Salmonella enterica*, by means of bacterial rapid detection using optical scattering technology (BARDOT) [21]. The data obtained showed a significant quantitative and qualitative difference in laser light scattering patterns for *S. enterica* suspensions grown in the presence of streptomycin and in control samples, clearly demonstrating the BARDOT applicability as a chemically independent method for the rapid study of the reaction of *S. enterica* on an antibiotic. In another study [22], the application of the laser light scattering method for the estimation of ABR of dangerous infections of *Bacillus anthracis, Yersinia pestis*, and *Burkholderia pseudomallei* was presented. Such application was carried out in real time by measuring the cell suspension laser light scattering intensity. It was determined that it is possible to distinguish between dividing (resistant) and not dividing (sensitive) organisms. The results were obtained in 4–10 h for *B. anthracis*, *Y. pestis*, and *B. pseudomallei*, showing a reduction of ABR analysis time by 50–75%, compared with conventional methods [23].

More recent methodology was developed for AST based on the BacterioScan 16R (Fisher, Waltham, MA, USA) laser light scattering technology, using methicillin resistance in *Staphylococcus aureus* and vancomycin resistance in enterococci as exemplars for important resistance phenotypes [24]. The concentration of cultures was 10^5^ CFU and the growth of organisms was observed in pure culture and in culture with the addition of an antibiotic. The analysis time was 60 min.

The examples [21,22,23,24] have shown the high potential of the LLS method and its ability to investigate microbiological samples within 1 to 4 h. Nevertheless, due to the cumbersome and expensive equipment for laser light scattering and its low availability, the method was not developed. At present, in connection with the development of new instrumental analytical technologies related to miniaturization [9,10,24], interest in the methods of LLS has resumed. Therefore, the search for ways to redesign this method for express analysis of ABR, especially in the MLOC format [25,26], seems to be very appropriate and relevant.

In this paper, the main features of miniaturized devices for laser light scattering analysis of bacterial suspensions have been considered both in static and dynamic signal recording modes, as well as ways of creating and integrating functional components for their implementation in a hybrid lab-on-a-chip format that will reduce the analysis time and provide mobility, which are important considerations for the further improvement of ABR express testing methods at points of care. A basic parameter for an analytical method for rapid testing of ABR is considered, namely the minimal time necessary for the estimation of microorganism viability, using *E. coli* strain M-17 as a model and a number of alternative species.

The transfer of the methodology to the capillary format, as well as comparison with the data of speckle optics dynamics, has been investigated. The multichannel format appropriate for a hybrid-integrated MLOC has been evaluated.

## 2. Materials and Methods

### 2.1. Microorganisms

A commercially available preparation of *Colibacterin siccum* was used, manufactured by Microgen Scientific and Production Association, Moscow, Russia (EAN code: 4605021000150, No. 94/161/279, 10 August 1994) containing the active substance—*E. coli* (*Escherichia coli*) strain M-17. The preparation was lyophilized by the manufacturer in a casein culture medium with the addition of a sucrose-gelatinous medium and contains 5 doses of a microbial mass of live microorganisms in one vial. One dose of the preparation contains at least 10^10^ CFU of *E. coli* M-17.

*Lactobacillus plantaum* (LBP) was obtained from the commercially available preparation Lactobacterin, manufactured by Microgen Scientific and Production Association (see above). A dose of preparation contains at least 10^9^ CFU of LBP.

*Bifidobacterium bifidum* (BFB) was obtained from a commercial preparation of Bifidumbacterin, manufactured by Microgen Scientific and Production Association (see above). A dose of preparation contains at least 10^7^ CFU of BFB.

*Stenotrophomonas maltophilia* was isolated from patients’ samples at St. Petersburg Pasteur Institute. Identification of the microorganism was made by MALI-TOF mass spectrometry.

### 2.2. Antibiotics

A sample of *E. coli* M-17 was examined for sensitivity to a basic set of antibiotics, listed in Table 1, using the Kirby–Bauer method [27]. Ceftriaxone, a cephalosporin antibiotic of III generation [28] with a broad spectrum of action, was selected for the LLS experiments. Ceftriaxone (for intramuscular and intravenous injection), 1 g, No. 1, Protech-CBM LTD, manufactured by PharmConcept LLC, Redkinsky Pilot Plant LLC, was used in all the LLS experiments with E. coli M-17.Bicillin, produced by LLC “Synthez” (Kurgan, Russian Federation). Gentamicin on discs RU No FCR 2009/06290 was used in other experiments.

### 2.3. Reagents

In all experiments with E. coli M-17, a standard meat-peptone broth from a two-phase system for aerobic and facultative anaerobic blood cultures (NICF 058104-200), produced by the LLC Research Center for Pharmacotherapy (NICF), St. Petersburg, Russia, was used as a nutrient medium. Phosphate buffer solution (PBS) was prepared by mixing aqueous solutions of Na_2_HPO_4_·12H_2_O and KH_2_PO_4_ in appropriate proportions to obtain pH 7.1 ± 0.1. In the LLS experiments on ABR assessment, ceftriaxone (for intramuscular and intravenous injections), manufactured by PharmConcept LLC, OAO Redkinsky Pilot Plant, was used. Ceftriaxone solutions of various concentrations in water for injections and phosphate buffer solution were prepared.

### 2.4. Laser Light Scattering Measurements

#### 2.4.1. Measurements of the Scattering Intensity in a Cylindrical Cuvette

**Figure 2:**
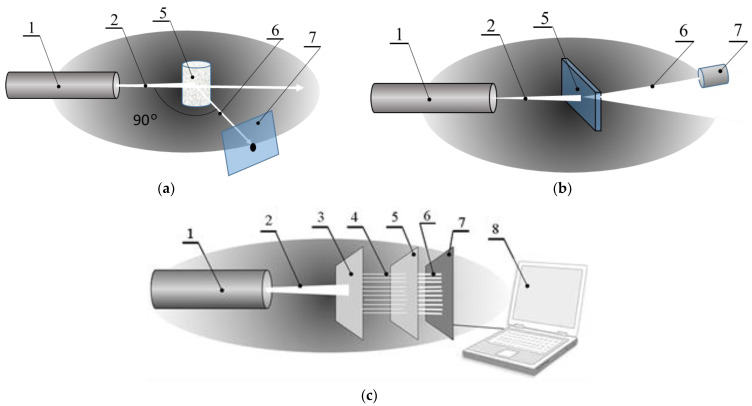
Schematic presentations of the LLS measurement setup: 90° scattering angle measurement, Scheme No. 1 (**a**); low-angle near-field speckle fluctuations capture, Scheme No. 2 (**b**); and multi-angle speckle fluctuation detection, Scheme No. 3 (**c**). 1—laser, λ = 645 nm; 2—laser beam; 3—collimator based on 2D diffraction grating; 4—multiple laser beam system; 5—multichannel chip; 6—a field of scattered light; 7—multichannel diaphragm coupled with a photodetector device; and 8—computer.

Measurements in a cylindrical cuvette were carried out using a Photocor Complex dynamic and static laser light scattering spectrometer (Photocor LTD, Moscow, Russia) (unit No. 1 in Figure 2a). The instrument made it possible to measure both the time function of scattered light intensity and the intensity autocorrelation functions in the automatic mode along with the temperature control of the sample. All the LLS measurements in a cylindrical cuvette were performed at a sample temperature of 37 °C and at a scattering angle of 90°. The wavelength of laser radiation is λ = 654 nm. The correlation functions data acquisition helped to obtain microorganisms size and to confirm that the scattered light intensity was attributed to microorganisms, which was important for studying the temporal dependence of the concentration of microorganisms in suspension.

It should be noted that the conventional accumulation of intensity correlation functions should be performed at a constant scattering intensity. These conditions are met only at a constant concentration of microorganisms after the cessation of the fission process. An increase in the concentration of dividing microorganisms and the corresponding increase in scattering intensity during signal accumulation inevitably leads to some distortion of the correlation function. A false contribution to the correlation function appears, representing the time span of signal accumulation but not the characteristic time of the diffusion of the microorganisms. However, a small difference in the correlation functions for dividing microorganisms and for microorganisms that have ceased fission is not a reliable indicator to detect the cessation of the microorganism fission.

#### 2.4.2. Measurements of Scattering Fluctuations in Planar Cuvettes

**Figure 3:**
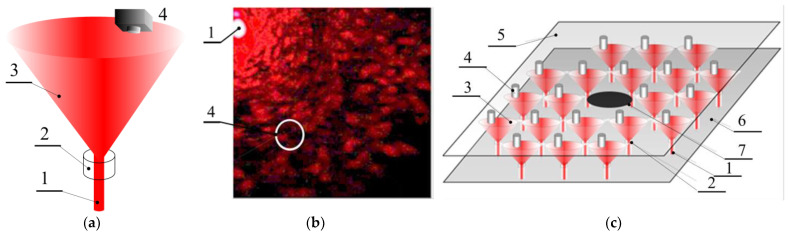
Schematic presentation of the principle of positioning of the detector unit (**a**,**b**) and multichannel ABR detection scheme using split laser beam (**c**). 1—laser beam, λ = 645 nm; 2—scattering volume; 3—scattered light field; 4—pin-photodiode; 5—cover of the multichannel chip; 6—base; and 7—shutter.

To study the kinetics of speckles in the near field of scattered laser light, the laboratory setups, as well as planar cuvettes and capillaries, were prepared at the Centre of Microtechnology and Diagnostics at St. Petersburg Electrotechnical University. The coherent semiconductor laser diodes with an optical power of CW = 30 mW and a wavelength of λ = 645 nm were used as incident light sources. The basic features of the optical setups used are presented in Figure 2b,c. No. 1 (as stated earlier) is the commercial instrument; No. 2 and No. 3 are laboratory setups. Units No. 2 and No. 3 contain a diaphragm with an aperture of 0.8 mm made of a black polymer film by laser ablation; lens with a focal length of *f* = 5 cm; a table for positioning a planar chip ad a semiconductor photosensitive pin-sensor (SPS) with an active diameter of 50 μm and input aperture of 2 mm; analogue-to-digital converter; and a computer. The SPS was placed on the periphery of the light spot generated by the transmitted laser beam. The size of the photodiode was chosen so that its surface area was overlapped by not more than three speckles at a time (Figure 3b,c). The distance L between the SPS surface and the scattering volume of sample was 50 mm; a CMOS sensor with a matrix of windows and a computer were evaluated as instruments for multichannel measurements of the speckle fluctuations as presented in Figure 2c and Figure 3a.

#### 2.4.3. Measurements of Scattering Fluctuations in Planar Cuvettes

**Figure 4:**
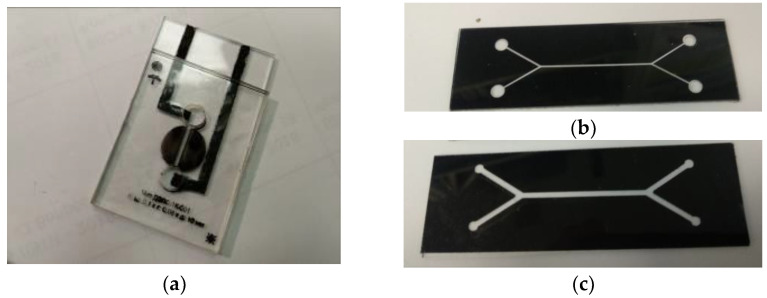
Sample capillary chips for LLS measurements: capillary (H × W × L) 0.1 mm × 1 mm × 10 mm (**a**); 0.08 mm × 0.7 mm × 30 mm (**b**); and 0.08 mm × 1.5 mm × 30 mm (**c**).

Experimental capillary chips applied in setup No. 2 were manufactured using flexible electronics or thick-film technologies. Polymethylmethacrylate plates, which were 1 mm thick and of optical quality, were used as a substrate and a lead. The glass plates were also used as a substrate. Polymer films of various thickness (80 ÷ 1000 μ), laser ablated to form a capillary system, were used as an intermediate layer for the profiling channels. The chips were baked or glued together to form sandwich structures. The typical dimensions of the measuring capillaries were (H×W×L): 1.0 mm × 1.0 mm × 10 mm; 0.08 mm × 0.4 mm × 30 mm (Figure 4).

**Figure 5:**
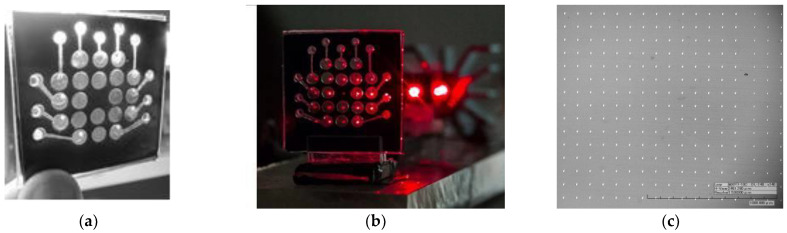
Experimental assembly of the matrix chip (**a**); a matrix chip in the installation illuminated by laser beams transmitted through the grating (**b**); and a micrograph of the diffraction grating from anodic alumina (**c**), where the window diameter is 10 μm and the distance between the windows is 100 μm.

A sample multichannel chip containing a matrix of measuring volumes (Figure 5a,b) was also made using the thick-film technology as a sandwich structure. A polyvinyl polymer film, 0.080 mm thick, Oracal 8500, with an adhesive layer (ORAFOL Europe GmbH, Germany), was used to form the channel profiles and to couple the vial structures. The topology of the channels was developed with the help of the AutoCAD programme and manufactured by precision laser ablation at Multitech, LLC (St. Petersburg, Russia).

The 2D diffraction gratings were made of anodic aluminum oxide at the Institute of Physics of the Academy of Sciences of Belarus (Minsk, Belarus) based on gamma-anodic aluminum oxide (Figure 5c). The diameter of the apertures is 0.01 mm, and the distance between them is 0.1 mm.

### 2.5. Basics of Static Laser Light Scattering by Particles Larger Than the Incident Light Wavelength and of the Speckle Fluctuation Kinetics in the near Field

When measuring the intensity of laser light scattering *I_s_* in bacterial suspensions, it is necessary to take into account that as the concentration of microorganisms increases, the contribution of, so-called, multiple scattering to *I_s_* increases. In the experiments conducted here, the size of microorganisms exceeds the wavelength of incidental light by 1.5…3 times. The angular distribution of *I_s_* is generally described by the Mie theory. However, it can be qualitatively described using the Rayleigh-Hans formulae [27]:

(1) $$I_{s}\sim {\left(n_{1}-n\right)}^{2}G^{2}(u)(1+\cos^{2}\theta ),$$

where:

(2) $$G(u)=\frac{3}{u^{3}}(\sin u-u\cos u),\;u=2x\sin\frac{\theta }{2},\;x=2\pi rn_{1}/\lambda ,$$

where: *θ* is the scattering angle, *n*_1_ and *n* are the refractive indices of scattering particle and medium, correspondingly, *λ* is the laser radiation wavelength, and *x* is the scattering parameter.

The larger the scattering parameter, *x*, and, correspondingly, the particle size, the more light that is scattered at small angles. This leads to an increasingly forward stretching scattering intensity angular distribution.

It is known that for *I*_s_ measurements in the multiple scattering regime with an elongated scattering intensity angular distribution, the contribution of multiple scattering is important mainly at small scattering angles and has lower effect on scattering at 90° [28,29]. Therefore, when measuring LLS signals for living microorganisms, only the intensity of laser light scattering at an angle of 90° was used. In this case, the intensity of light scattered by microorganisms could be considered proportional to their concentration.

Speckles are the result of the interference of light waves scattered by sample inhomogeneities. The scattered waves differ in phase and, after summation on the surface of the detector, give an interference pattern with amplitude and position distributed randomly.

Speckles can be generated in the far field (the Fraunhofer scattering region) and in the near field (the Fresnel scattering region), depending on the Fresnel number:

(3) $$F=\frac{d^{2}}{L\lambda },$$

where: *d* is the diameter of the laser beam, *L* is the distance between the scattering object and the surface of the detector, and λ is the wavelength of the incident light.

The far-field conditions are satisfied at *F* << 1, while the near-field conditions are satisfied at *F* > 1. For *F* >> 1, the approximation of geometrical optics is applicable.

The properties of far-field speckles depend only on the shape and size of the scattering volume. For example, the average speckle size (*s*) is determined by the values of *d*, *L*, and λ so that:

(4) $$s=\lambda \frac{L}{d},$$

Speckles of the near field, on the other hand, are related to the properties of the scattering object. In this paper, the kinetics in the near field were measured in a cylindrical cuvette with a diameter of 8 mm and in planar cuvettes.

In a multichannel chip, fluctuations were monitored for *E. coli* M-17 suspension in aqueous suspension and in a solution of ceftriaxone. The size of the speckle spot and the frequency of the fluctuations correlate with the condition of microorganisms in suspension. The speckle capture arrangement is presented in Figure 2c.

## 3. Results and Discussion

### 3.1. Characterization of E. coli M-17 Using Disc-Diffusion Kirby-Bauer ABR Profiling

The ABR for a sample of *E. coli* M-17 was profiled using the classical disc-diffusion Kirby–Bauer method (at St. Petersburg Pasteur Institute) in Petri dishes. The results presented in Table 1 show that the most representative pharmacological group of antibiotics in this study was the latest generation cephalosporins, which demonstrated efficacy for 87% of samples, and 13% showed a moderate efficacy in a total of 15 samples. Therefore, for laser light scattering ABR analysis, ceftriaxone, an antibiotic of III generation cephalosporins, was chosen. The bactericidal effect of this antibiotic is realized by inhibiting cell wall synthesis in microorganisms. Ceftriaxone acetylates membrane-bound transpeptidases, thus violating the cross-linking of peptidoglycans necessary to ensure the strength of the cell wall of this Gram-negative microorganism. It is known that the drug is active against Gram-negative aerobes, including *E. coli* M-17.

### 3.2. Fission of Microorganisms Monitoring with LLS

**Figure 6:**
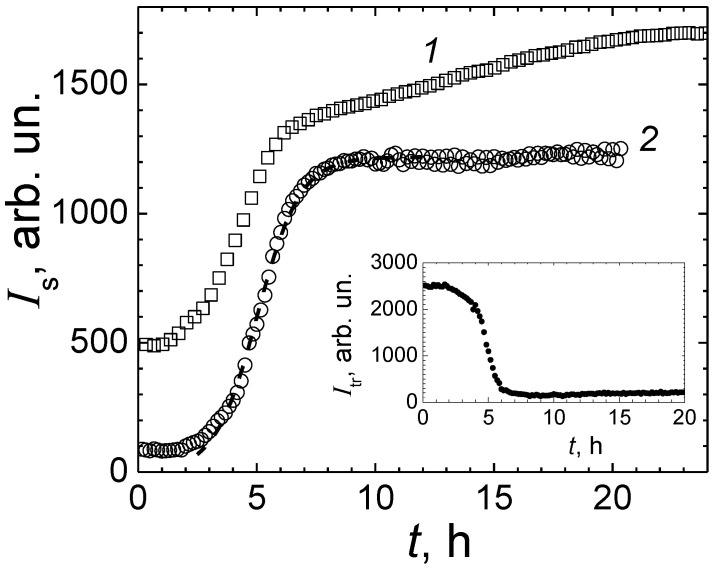
Dependences of the laser light scattering intensity on time for *E. coli* M-17 in meat-peptone broth. 1—sample No. 3 isolated from the air intake in sealed vial; 2—sample No. 4 with unlimited supply of air in an open vial. Dashed line is the data fit to the Verhulst formula. Insert: The intensity of light transmitted through sample with unlimited airflow as a function of time.

Two samples of *E. coli* M-17 numbered No. 1 and No. 2 were prepared by dissolving the dry preparation of *Colibacterin Siccum* in water for injections, followed by gentle shaking for 30 min to receive stock suspensions of concentrations approximately 1.7 × 10^5^ CFU/mL (CFU—colony-forming unit) and 2.7 × 10^4^ CFU/mL correspondingly. Then, 200 μL of each suspension in water was added to 2.0 mL of the nutrient broth to finally obtain suspensions of 1.5 × 10^4^ CFU/mL (sample No. 3) and 2.4 × 10^3^ CFU/mL (sample No. 4). The measurements on each sample were performed sequentially after sample had been prepared in the cylindrical glass cell (vial). Sample was placed into the thermostat adjusted to the temperature of 37 °C. Then, 10 min later, the process of measurements was started with the Photocor Complex spectrometer. These preliminary measurements of scattering intensity during population growth were carried out under aerobic and anaerobic conditions (Figure 6). Thus, in sample No. 3, the vial cap was sealed and the air reserve was limited. In sample No. 4 the vial cap was open and the inflow of air was unlimited. In both samples, the volume of the nutrient broth was 2.0 mL.

The intensity of light scattered by microorganisms suspended in nutrient broth is proportional to their concentration. An increase in the intensity of scattering is an indicator of the process of dividing microorganisms, which, in turn, is the most important sign that they are viable. The intensity of laser light scattering for samples No. 3 and No. 4 containing *E. coli* M-17 suspension in meat-peptone broth, as shown in Figure 6, shows that during the first 2–2.5 h of the experiment the scattering intensity remains almost constant, exhibiting a lag time in the fission of microorganisms presumably caused by shock after the dissolution of dry Colibacterin preparation in water and by transfer of microorganisms to a new medium nutrient broth. After this initial adaptation period for sample No. 4 with unlimited inflow of air, the dependence of the scattering intensity on time fits well with a curve based on the classical Verhulst formula, taking into account a 2.6 h initial lag time in the *E. coli* M-17 fission process. Approximately 7 h after the start of the run with sample No. 4, the laser light scattering intensity saturates, arriving to the limiting concentration *c*_max_ of *E. coli* M-17 in the medium with limited nutrient supply. The following parameters were obtained based on the data fit to the Verhulst formula: the initial value *I*_s_ = 80 arb. units, asymptotic limit *I*_max_ = 1.2 × 10^3^ arb. units, and relative growth rate *r* = 1.3 h^−1^.

The intensity of laser light scattering, *I*_s_, in sample No. 3 has a complex time dependence with the observed second stage of population growth, attributed to the interchange between aerobic and anaerobic regimes of *E. coli* fission. In our study, a salient point on the characteristic time dependence of the intensity of scattering serves as an indicator of the antimicrobial effect of the antibiotic. All further experiments were performed only in the aerobic regime to avoid unnecessary complications in the interpretation of data caused by the interchange between the aerobic and anaerobic regimes of the *E. coli* M-17 fission process.

The intensity, *I*_tr_, of light transmitted through a 14 mm thick sample (the internal diameter of the vial) was measured simultaneously with the scattering intensity, *I*_s_. These data are shown for sample No. 4 (aerobic conditions) in Figure 5, inset. While the intensity of the scattered light increases and arrives to the limiting value, the transmitted light intensity *I*_tr_ decreases by more than an order of magnitude. For such a significant decrease, a significant multiple scattering contribution to transmitted light intensity, *I*_tr_ is expected, which makes the interpretation of laser light scattering and turbidimetry data essentially complicated. In a single laser light scattering regime, the transmitted light intensity *I*_tr_ dependence on the concentration of microorganisms should obey the Bouguer–Lambert–Beer law, and the scattering intensity, *I*_s,_ should be proportional to the concentration of microorganisms, which corresponds to a linear dependence of the logarithm of *I*_tr_ on *I*_s_.

**Figure 7:**
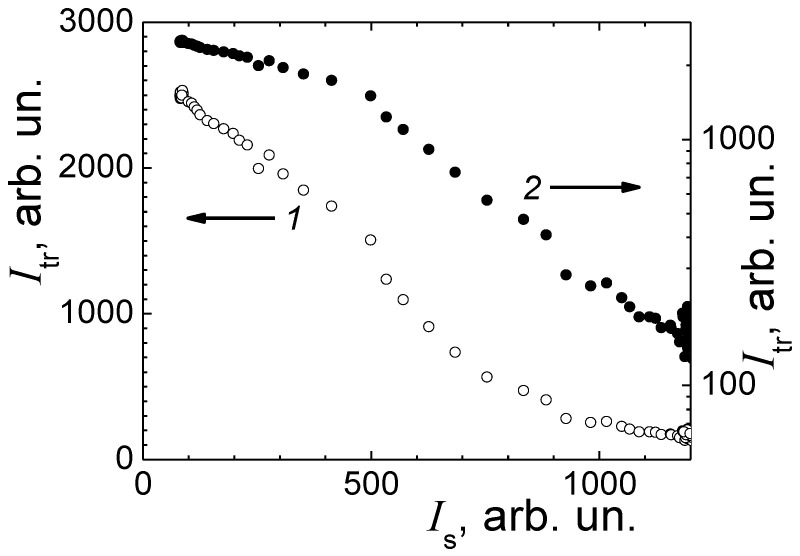
Dependence of the intensity *I*_tr_ of the transmitted laser beam on the scattering intensity *I*_s_ in linear (1) and semi-log (2) scales.

In Figure 7, a joint analysis of the transmitted laser beam intensity, *I*_tr_, and the scattering intensity, *I*_s_ is presented. Two line segments are obtained with a salient point between them, and their different slopes are obtained in a semi-log plot. For a low concentration of microorganisms and low laser light scattering intensity *I*_s_, the data corresponds to the Bouguer–Lambert–Beer law, and the slope of this line segment is determined by the extinction coefficient. The next line segment with a larger slope is distinctive for the regime of multiple scattering. An interchange between the modes of single and multiple scattering of light is presented in the study [25,26] of multiple scattering for light propagating along the axis in a long cylinder.

In this study, the microorganism size exceeds the wavelength of visible light; for bacterial suspension, the scattering intensity radial distribution function is stretched forward. The light is most intensively scattered at small angles in a narrow cone. This geometry is similar to the geometry of a narrow long cylinder used in [30]. Thus, it is necessary to take into account multiple scattering in the interpretation of *I*_tr_ data at a high concentration of *E. coli* M-17 in sample No. 4.

When the radial distribution function is largely extended forward, the direction of scattering at the angle of 90° is outside the cone of intensive small-angle scattering. At the scattering angle of 90°, the contribution of multiple scattering to intensity *I*_s_ is less than this contribution to the scattering intensity at small angles [30]. Lack of any noticeable deviations of *I*_s_ from the Verhulst’s dependence at a high concentration of microorganisms shown in Figure 7 also confirms the insignificance of multiple scattering contribution to the intensity of scattering *I*_s_ in our experiments. The dependence of *I*_tr_ on *I*_s_ in Figure 7 resembles the dependence of *I*_tr_ on the concentration in an optically thick sample. Therefore, we neglect the contribution of multiple scattering and consider that the scattering intensity *I*_s_ is proportional to the concentration of *E. coli* M-17 under the conditions of our experiments.

### 3.3. Cessation of Microorganisms’ Fission by Ceftriaxone Monitoring with LLS

The *E. coli* M-17 suspension with population grown, as presented in Figure 6, at maximal concentration *c*_max_ in sample No. 4, enabled generation of a stable suspension No. 5, which was used for the preparation of samples No. 6 and No. 7. This helped avoid a loss of time in the initial stage of growth, caused by the transition from the dry form of the preparation to the suspension of *E. coli* M-17. Sample No. 6 was prepared by diluting 0.5 mL of live *E. coli* suspension No. 5 in 2.0 mL of nutrient broth. For sample No. 7, 1.0 mL of suspension No. 5 was diluted in 2.0 mL of nutrient broth. The small volumes of concentrated solution of ceftriaxone in water for injections were added to *E. coli* M-17 suspensions in samples No. 6 and No. 7 at fixed times to obtain antibiotic concentrations of 0.84 mg/mL and 0.070 mg/mL, respectively.

**Figure 8:**
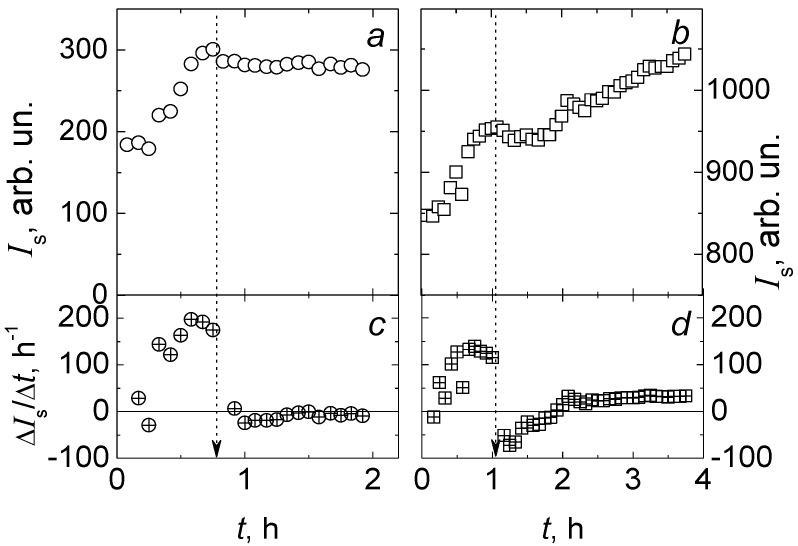
Effect of ceftriaxone on *E. coli* fission in nutrient broth for samples No. 3 and No. 4 with ceftriaxone concentrations: (**a**)–0.84 mg/mL and (**b**)–0.070 mg/mL, respectively. Dependences of laser light scattering intensity on time (**a**,**b**) and the slopes of these dependences (**c**,**d**). The arrows indicate the time of ceftriaxone injection in samples.

The effect of ceftriaxone added to the suspension of live *E. coli* M-17 is shown in Figure 8 for samples No. 6 and No. 7, which differ by an order of magnitude for antibiotic concentrations. Monotonous increase in laser light scattering intensity, *I*_s_, was monitored over a period of 40 to 60 min, and the fission process of *E. coli* M-17 was clearly detected. Then, small volumes of ceftriaxone solution were injected into samples No. 6 and No. 7. This injection of ceftriaxone solution caused a slight decrease in the concentration of microorganisms, which was noticeable (Figure 8).

In sample No. 6, the *E. coli* M-17 fission (Figure 8a) stopped completely almost immediately after the addition of the antibiotic. In sample No. 7 (Figure 8b), where the antibiotic concentration was an order of magnitude lower, *E. coli* M-17 fission continued, but the slope of the time dependence of the scattering intensity *I*_s_ apparently decreased, indicating a decrease in the fission rate of microorganisms caused by an antibiotic—ceftriaxone. An abrupt change in the slope of the *I_s_* dependence on time is shown in Figure 8c,d.

### 3.4. The Minimal Time Estimation for Rapid Assessment of Viability for E. coli M-17 Using Classical Laser Light Scattering in a Cylindrical Cell

For comparison of the method of laser light scattering with other methods, it is important to estimate the minimal time required for determining ABR with measurements of laser light scattering intensity. The estimated random error in laser light scattering intensity is 1–2%. Therefore, for reliable detection of population growth with scattering intensity measurements, it is necessary that the intensity increases in the experiment, at least, by no less than ten percent. For this purpose, it is necessary to provide an adequate nutrient supply in the broth, that is, a large capacity of the ecological niche for these microorganisms. For instance, after dilution of lyophilic Colibacterin in water, a significant increase in scattering intensity had not been observed, despite the nutrients included in the dry preparation according to description.

The time spent on recording the growth of the scattering intensity by ten percent depends on the fission rate of microorganisms, the value quantitatively expressed by the relative growth rate *r* in the Verhulst’s differential equation. The integral Verhulst’s logistic curve exhibits the increase in microorganism concentration *c* from the initial value *c*_0_ to the final maximum concentration *c*_max_. The time of colony growth for small concentration increments ∆*c* = *c* − *c*_0_ is obtained in the finite-difference Equation:

(5) $$\Delta t=\frac{1}{r\left(1-\frac{c}{c_{\max}}\right)}\frac{\Delta c}{c},$$

subject to inequality ∆*c/c* << 1.

The time of concentration increase by 10%, which corresponds to ∆*c/c* = 0.1, is the estimate of the minimal time required for detection of microorganism fission, confirming their viability. Assuming a constant value of ∆*c/c*, we obtain that minimal ∆*t* is achieved at *c/c*_max_ << 1, namely at low concentration *c* in comparison with *c*_max_.

An initial concentration increase corresponds to a relatively low increase in ∆*t*. Arriving to concentration *c* = *c*_max_/2, the value of ∆*t* is doubled. However, the following concentration increase is accompanied with a rapid increase in ∆*t* caused by low residual 1 − *c/c*_max_. Therefore, it is recommended to perform experimental ABR detection at concentration *c* < *c*_max_/2. Hence, the range of 0.1 *r*^−1^ < ∆*t* < 0.2 *r*^−1^ corresponds to a 10% concentration increment. For the experimental value of *r* = 1.3 h^−1^, the ∆*t* range is 5–10 min. The minimum necessary time for detection of cell fission process cessation could not be shorter than ∆*t*. The estimate of the minimal time for the whole experiment of ABR detection is approximately 2∆*t*, which corresponds to 10–20 min. This time is the sum of the minimum time for cell fission process detection and of the minimum time for detection of fission process cessation or slowdown by antibiotic injection.

### 3.5. Towards LLS in Lab-on-A-Chip Format

#### Measuring the Kinetics of Speckles in a Cylindrical Cuvette and in a Capillary Chip

For a comparative study of the characteristic time of the recorded processes in dynamic colloidal systems developing in a large volume and in the capillary, the measurements were performed first on the *E. coli* M-17 model. The intensity fluctuations occurred due to Brownian motion, sedimentation, and changes in particle mobility due to aggregation caused by cell structure degradation, fission, or death of microorganisms. In addition, due to the small input aperture of the photodetector device, the sensitive area of which corresponds to the characteristic size of 1…3 speckles (Figure 3a,b), spatial averaging of the fluctuations does not occur, thus increasing the sensitivity of the method, which could be called micro-dynamic turbidimetry (μDTD). The method of μDTD allows recording of the change in the state of the suspension at the earliest stages of the process to be monitored, when conventional methods of turbidimetry do not provide sufficient accuracy.

**Figure 9:**
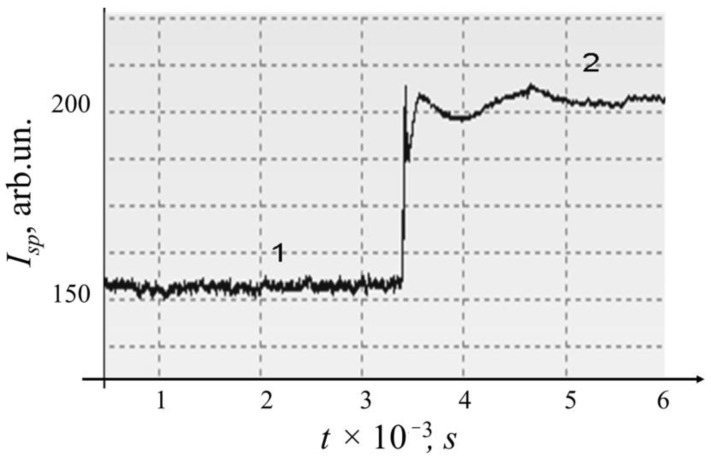
The change in intensity of fluctuations of *I*_sp_ speckles for samples of *E. coli* M-17 No. 2 (1) and *E. coli* M-17 with ceftriaxone No. 7 (2).

It is important to note that in a capillary cell, as well as in a cylindrical cell, the fluctuations in the intensity of the scattered light are an indicator of the reaction in sample, while the average value of intensity remains mostly unchanged. Moreover, when compared with a cylindrical cuvette, the amplitude of fluctuations in the capillary cuvette is lower (both during the reaction and in the reference sample), which is associated with a decrease in the path of the laser beam through sample. Acceleration of processes in the capillary as compared with the cylindrical cell is associated with the possibility of an increase in the concentration of the suspension. A result of the kinetics of intensity, *I*_sp_ fluctuations of speckles for *E. coli* M-17 suspension in the absence and presence of ceftriaxone antibiotic in the cylindrical cuvette were observed for sample No. 2 (Figure 9). The addition of a small volume of antibiotic solution led to an increase in the average level of signal, which is connected with the features of the scattering at low angles. The amplitude of the intensity fluctuations, *I*_sp_, of speckles in contrast was reduced. The effect manifested itself immediately on the addition of an antibiotic in the same manner as in Figure 6.

Speckles are a result of the interference of light waves scattered by sample inhomogeneities. The scattered waves differ in phase and, after summation on the surface of the detector, give an interference pattern with amplitude and position distributed randomly. Measurements of the kinetics of fluctuations were carried out in the near field (*F* >>1, Equation (3)) and at low angle as shown in Figure 3b. Since the intensity fluctuations occur due to Brownian motion and mobility caused by fission of microorganisms, and these processes were reduced by the effect of antibiotic, Figure 9 shows the reduction of fluctuation intensity that was observed at the first stage of antibiotic exposure in the cylindrical cell.

**Figure 10:**
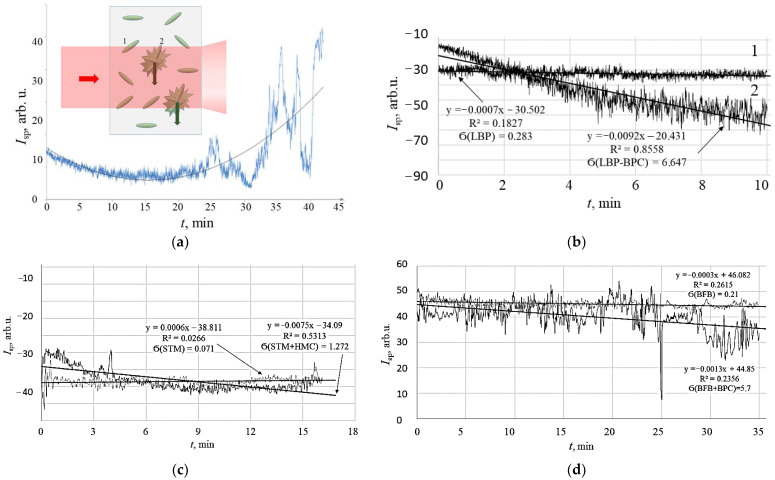
Time dependence of speckle fluctuation kinetics, *I*_sp_ for suspensions of microorganisms exposed to antibiotics, observed in a planar cell, *d* = 1 mm. (**a**) Schematic representation of bacterial agglomerates migration direction in the cell (insert), and observation of *E. coli* M-17 suspension exposed to ceftriaxone; (**b**) *Lactobacillus plantarum* (LBP), strain 8P-A3 (1), 10^7^, LBP with benzyl penicillin (BPC) (2); (**c**) *Stenotrophomonas maltophilia* (STM), 10^7^ CFU/mL (1), STM with gentamicin (GMC) (2); and (**d**) *Bifidobacterium bifidum* (BFB) (1), 10^7^ CFU/mL, BFB and ceftriaxone (CTA) (2). Standard deviation data are calculated for time ranges 5 to 10 min.

Samples were also investigated in planar capillary cells. The scattering of sample No. 2 of *E. coli* M-17 with 20 mg/mL ceftriaxone was observed in the 1 mm thick planar cell (Figure 10). The pattern shows some initial decrease in the activity of bacteria in suspension, which started immediately and lasted for approximately 14 min after adding the antibiotic. In the next phase of the antibacterial process, the increase in intensity fluctuations of speckles was observed. The last stage showed the massive destruction of the suspension with the formation of large aggregates and their sedimentation (Figure 10, insert).

The viability of microorganisms was also observed for *Lactobacillus plantarum* (LBP), *Stenotrophomonas maltophilia* (STM) and *Bifidobacterium bifidum* (BFB) in pure samples without antibacterials (1 in Figure 10b–d) and in the presence of antibiotics (2 in Figure 10b–d). The observation time was 10, 15 and 35 min, correspondingly. The constant average level of the signal and amplitude of fluctuations was observed for pure samples without antibacterial preparations (signal 1). Under the exposure of antibiotics, the decline of the average level of the signal was observed simultaneously with the growth of the amplitude of fluctuations (signal 2). The results are summarised in Table 2.

Results in Table 2 show that, under exposure to active antibiotics, the speckle fluctuations increase their amplitude, and the average value slightly decreases. In the format, as shown in Figure 10a (insert), when sedimenting agglomerates of bacteria and debris cross the incidental laser light radiation, the action of an antibiotic manifests itself in increasing fluctuations. The standard deviation of the fluctuations differs considerably relating the “pure” samples, namely up to 5%, which is sufficient to make conclusions.

### 3.6. Observation of Intensity Fluctuations of Speckles in a Multichannel Matrix Cell

The multichannel setup of express ABR testing is necessary to monitor an array of basic lines of antibiotics. The setup studied in this paper for multichannel micro-dynamic turbidimetry (MμDTD) is shown in Figure 3c. An experimental chip containing a matrix of detection volumes is presented in Figure 5a,b.

**Figure 11:**
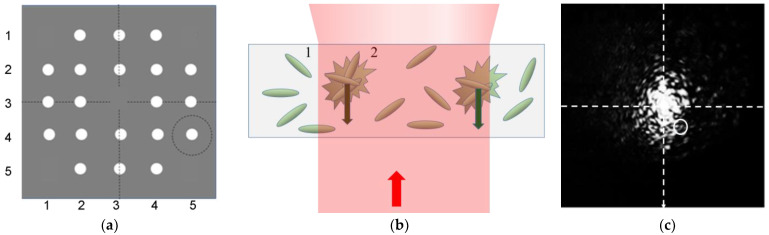
Registration of suspensions of microorganisms in a matrix chip using a 2D collimator (**a**) for the array of 20 measuring cuvettes, an optical scheme of the speckle excitation in microcells (**b**), where 1–microorganism, 2–cluster of microorganisms, and the speckle field of the cuvette of the *E. coli* M-17 filled in phosphate buffer (**c**), where the white circle indicates the signal reception aperture.

In a multichannel arrangement, the virtual “pin-sensor” (sensing area on the CMOS matrix) should also be positioned on the periphery of the incidental light spot generated by the transmitted split laser beam (Figure 11b). The size of the sensor was chosen in such a way that its surface area was overlapped by not more than three speckles at a time (Figure 11c). The distance L between the SPS surface and the scattering volume of sample is 50 mm.

Qualitative observations of the kinetics of speckles of scattered laser radiation in microcells containing *E. coli* M-17 microorganisms dispersed in the liquid phase at a concentration of 5 ⋅ 10^3^ CFU/mL in sodium phosphate buffer were performed. Changes were also observed when ceftriaxone was added to the suspension at a concentration of 1.0 mg/mL.

**Figure 12:**
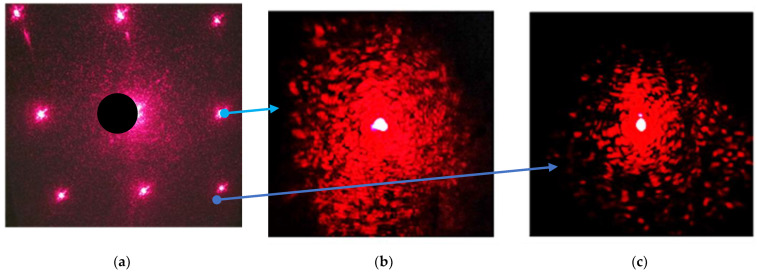
Speckle fields generated by *E. coli* M-17 suspension No. 2 loaded in microvials of multichannel planar chip with 8 vials after 10 min of observation (**a**); speckle fields generated by the suspension of *E. coli* without an antibiotic (**b**); and the same as b with ceftriaxone, 20 mg/mL (**c**). Arrows show the enlarged images of the corresponding cells.

At the first stage, eight cells (Figure 4a) were filled with *E. coli* M-17 suspension, and the uniformity of scattering by bacteria was determined. An example of a scattering pattern in one of the peripheral cells is shown in Figure 12b in the pure sample of *E. coli.* In the cell filled with suspension No. 2 containing ceftriaxone at 10 min, the initial speckle pattern degradation is shown in Figure 12c. A decrease in the scattering intensity is noticeable.

**Figure 13:**
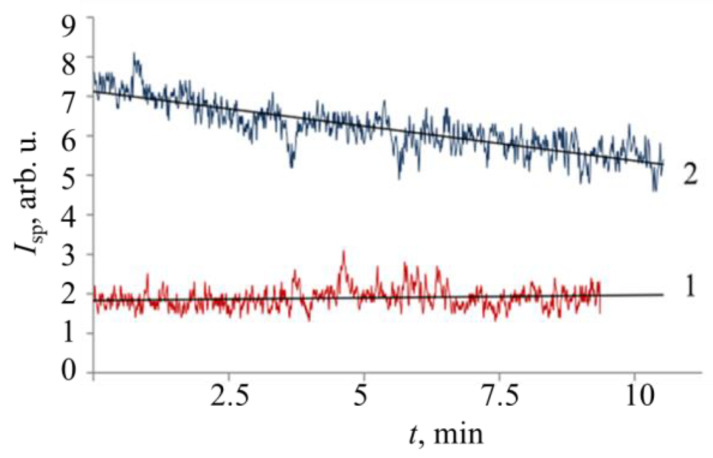
Time dependencies of scattering intensity fluctuations, *I_sp_* measured in the cell of planar multichannel chip (Figure 12) for the suspension of E. coli M-17, c = 10^8^ CFU/mL (1) and the same with the addition of ceftriaxone (2) at the concentration of 1.0 mg/mL.

Thus, the suspension of bacteria in the cell shows stable behaviour for at least 10 min from the start of the signal recording (1 in Figure 13), while sample with an antibiotic shows a monotonous decrease in the average intensity of speckle fluctuations, *I*_sp_ (2 in Figure 13). The signals were recorded with the help of a CMOS sensor.

## 4. Conclusions

Various modes of laser light scattering in the detection of the viability of microorganisms under exposure to antibiotics were investigated in order to select the format most appropriate for the implementation of MLOC. A classical format of measurements in a cylindrical cuvette at a 90° scattering angle and analysis of fluctuations of speckles at a low angle in a cylindrical cuvette and in planar capillary chips were compared using *E. coli* M-17 (Gram-negative facultative anaerobe) and ceftriaxone, *Lactobacillus plantarum* (Gram-positive anaerobe) and Benzyl penicillin, *Stenotrophomonas maltophilia* (Gram-negative aerobe) and Gentamicin and *Bifidobacterium bifidum* (Gram-positive anaerobe) and ceftriaxone.

LLS of the suspension in meat-peptone broth demonstrated an increase in the intensity of scattering at a 90° angle, showing the process of microorganism fission and a population increase of 15 times in 7 h. Sample was used for further testing of viability with the classical instrument Photocor. The effect of ceftriaxone added at the concentration of 1.0 mg/mL to the suspension of fissile *E. coli* M-17 demonstrated a complete termination of fission and corresponding constant scattering intensity, *I*_s_. The time needed for the evaluation of the effect of antibiotics on the culture of living microorganisms was evaluated as 20 min.

Measuring the kinetics of speckles in a cylindrical cuvette and in a planar capillary chip was compared. In the vertical geometry of measurements, intensity fluctuations increase, which occur due to Brownian motion, sedimentation, and the changes in particle mobility due to aggregation caused by the recognition of antigen particles, fission, or death of microorganisms. In addition, due to the small input aperture of the photodetector device, the sensitive area of which corresponds to the characteristic size of 1…3 speckles, spatial averaging of the fluctuations does not occur, which increases the sensitivity of the method. This allows recording of the change in the state of the suspension at the earliest stages of the process, when conventional methods of turbidimetry do not provide sufficient accuracy.

The data obtained in the cylindrical cuvette demonstrated an increase in the average level of light at a low scattering angle after the addition of antibiotic. This effect is opposite to the effect observed at a 90° scattering angle, where the light intensity lowers with the decrease in concentration because the straight direction of light detection causes some extent of mixing between incidental light and the speckles, which yield an increase in the average level of light. The effect of an antibiotic at 1.0 mg/mL was also immediate but showed the reduced amplitude of speckle fluctuations, which may indicate the reduction of activity of microorganisms. In the reference sample, the average value of the scattered light intensity did not change. It follows that the measurements of intensity fluctuations provide more information than the measurements of the average intensity value.

It has been shown that the viability of microorganisms under antibiotic exposure can be estimated in 10 to 30 min using the intensity of laser light scattering in cylindrical cuvette.

Further studies were carried out in miniature capillary planar devices, formed on polymethyl methacrylate (PMMA) and glass substrates. The results were associated with the possibility of using suspensions of higher concentrations, as well as a reduction of stray light and multiple scattering. Observation of temporal changes in the pattern of speckles obtained for samples of microorganisms showed that for samples exposed to antibiotics the speckle intensity increased in the vertical arrangement of detection. Durable experiments with *E. coli* M-17 demonstrated the development of the bactericidal process in time, which started with a gradual decrease in average scattering intensity and the growth of the fluctuation amplitude, which lasted for approximately 15 min. In the next stage, the fluctuations started to increase dramatically, which could be attributed to the formation of large aggregates of dead cells and cell debris. The informative time for observing the viability decrease could be estimated as 10–15 min on the basis of the *E. coli* M-17 model. The experiments with the other three microorganisms in capillary cells demonstrated that ABR testing could be completed using LLS within 10 min using the amplitude of speckle fluctuations in near-field format as a detection principle. The next stage towards MLOC implementation was the observation of viability using a multichannel format, and the interference grating had been pretested. Comparison of speckle fluctuations in the reference sample with the one containing ceftriaxone with *E. coli* M-17 in the horizontal position of the microfluidic system with eight cells shows that, in the first case, the fluctuations were stable, while in the case of antibiotic presence, a decline of the scattering pattern was observed.

It has been shown that the laser light scattering technique is an efficient tool for detecting the viability of microorganisms. The time of detection decreased to 10 min using the capillary system. The latter also has an advantage of sample volume reduction, making parallel experiments convenient for automation for a rapid evaluation of ABR in culture for 10–20 min and integration into hybrid microbiological systems for express total analysis. This will significantly speed up the search for an effective antibiotic and help to determine the necessary concentration for it in comparison with traditional methods.

## Figures and Tables

**Table 1:** Sensitivity of sample of *E. coli* M-17 to a basic set of antibiotics.

Name of Antibiotic	Response of Microorganism to Antibiotic Exposure			Pharmacological Group
	S ^1^	MR ^2^	R ^3^	
Amoxicillin, Ampicillin	+			Penicillins
Amoxicillin/clavulanic acid, Ampicillin/Sulbactam, Tricarcillin/Clavulanate (Timentin)	+			Penicillins combined with beta-lactamase inhibitors
Tetracyclin, Dixiciclin	+			Tetracyclines
Imipenem, Meropenem	+			Carbapenems
Levomicetin	+			Amphenicols
Levoflixacin, Lomeflixacin, Moxifloxacin, Norfloxacin,	+			Quinolones/fluoroquinolones
Ceftazidime, Ceftibuten, Ceftriaxone *, Cefuroxime,	+			Cephalosporins
Vancomycin		+		Glycopeptides
Azithromycin, Clarithromycin, Erythromycin		+		Macrolides and Azalides
Tobramycin		+		Aminoglycosides
Carbenicillin		+		Penicillins
Bacitracin			+	Polypeptide antibiotic
Benzylpenicillin, Oxacillin			+	Penicillins
Gemifloxacin			+	Fluoroquinolones
Clindamycin			+	Lincosamides
Linezolid			+	Oxazolidonones
Neomycin, Streptomycin			+	Aminoglycosides
Oleandomycin, Roxithromycin, Tylosin, Novobiocin **			+	Macrolides and Azalides
Trimethoprim/sulfamethoxazole, Fosfomycin, Fusidine			+	Other antibiotics

S ^1^—Sensitive; MR ^2^—Moderately resistant; R ^3^—Resistant; * the antibiotic used in the experiment; and ** close to macrolides.

**Table 2:** Observation of speckle fluctuations parameters for suspensions of microorganisms with and without antibiotics.

Microorganism	C, CFU	Antibiotic	C, mg/mL	Ϭ	R^2^
*E. coli* M-17	2.6 × 10^4^	Ceftriaxone	1.0	0.8	-
*Lactobacillus plantarum*	10^7^	-	-	0.283	0.183
		Benzyl penicillin	0.20	6.65	0.86
*Stenotrophomonas maltophilia*	10^7^	-	-	0.071	0.027
		Gentamicin	1.0	1.27	0.53
*Bifidobacterium bifidum*	10^7^	-	-	0.21	0.026
		Ceftriaxone	1.0	5.7	0.236

## Data Availability

Not applicable.
